# Molecular Characterization of Extended-Spectrum β-Lactamase–Producing *Escherichia coli* and *Klebsiella pneumoniae* Among the Pediatric Population in Qatar

**DOI:** 10.3389/fmicb.2020.581711

**Published:** 2020-11-11

**Authors:** Andres Perez-Lopez, Sathyavathi Sundararaju, Hassan Al-Mana, Kin Ming Tsui, Mohammad Rubayet Hasan, Mohammed Suleiman, Mohammed Janahi, Eman Al Maslamani, Patrick Tang

**Affiliations:** ^1^Division of Microbiology, Department of Pathology, Sidra Medicine, Doha, Qatar; ^2^Weill Cornell Medical College in Qatar, Doha, Qatar; ^3^Biomedical Research Centre, Qatar University, Doha, Qatar; ^4^Division of Infectious Diseases, Faculty of Medicine, University of British Columbia, Vancouver, BC, Canada; ^5^Division of Pediatric Infectious Diseases, Sidra Medicine, Doha, Qatar

**Keywords:** children, whole-genome sequencing, *E. coli*, *K. pneumoniae*, extended-spectrum β-lactamases, CTX-M-15, fluoroquinolones

## Abstract

**Introduction:**

Although extended-spectrum β-lactamase (ESBL)–producing Enterobacterales are a public health problem in the Arabian Peninsula, data on the molecular characteristic of their antimicrobial resistance determinants in children is limited.

**Aim:**

To determine the molecular characteristics of ESBL-producing *Escherichia coli* and *Klebsiella pneumoniae* in the pediatric population of Qatar.

**Methods:**

Whole-genome sequencing was performed on ESBL-producing *E. coli* and *K. pneumoniae* isolates recovered from screening and clinical specimens from pediatric patients at Sidra Medicine in Doha from January to December 2018.

**Results:**

A total of 327 ESBL producers were sequenced: 254 *E. coli* and 73 *K. pneumoniae*. Non-susceptibility rates to non-β-lactam antibiotics for both species were 18.1 and 30.1% for gentamicin, 0.8 and 4.1% for amikacin, 41.3 and 41.1% for ciprofloxacin, and 65.8 and 76.1% for cotrimoxazole. The most common sequence types (STs) were ST131 (16.9%), ST38 and ST10 (8.2% each) in *E. coli* and ST307 (9.7%), and ST45 and ST268 (6.9% each) in *K. pneumoniae*. CTX-M type ESBLs were found in all but one isolate, with CTX-M-15 accounting for 87.8%. Among other β-lactamases, TEM-1B and OXA-1 were coproduced in 41 and 19.6% of isolates. The most common plasmid-mediated quinolone resistance genes cocarried were *qnr A/B/E/S* (45.3%). Ninety percent of gentamicin non-susceptible isolates harbored genes encoding AAC(3) enzymes, mainly *aac(3)-IIa*. Only two of 57 isolates harboring *aac(6′)-Ib-cr* were non-susceptible to amikacin. Chromosomal mutations in genes encoding DNA gyrase and topoisomerase IV enzymes were detected in 96.2% fluoroquinolone-non-susceptible *E. coli* and 26.7% fluoroquinolone-non-susceptible *K. pneumoniae*.

**Conclusion:**

Our data show that CTX-M enzymes are largely the most prevalent ESBLs in children in Qatar with a predominance of CTX-M-15. Carbapenem-sparing options to treat ESBL infections are limited, given the frequent coproduction of OXA-1 and TEM-1B enzymes and coresistance to antibiotic classes other than β-lactams.

## Introduction

Over the last four decades, extended-spectrum β-lactamases (ESBLs) have presented a major challenge to treating infections caused by Enterobacterales in health care and community settings (Peirano and Pitout, 2019). Resistance to third- and fourth-generation cephalosporins mediated by ESBLs and the often associated coresistance to other antibiotic classes, such as fluoroquinolones, aminoglycosides, and sulfonamides, have led to the increased use of carbapenems worldwide, which in turn is linked to increasing resistance to these agents (Peirano and Pitout, 2019). Currently, the global epidemiology of ESBLs is dominated by CTX-M type enzymes, mainly due to the explosive dissemination of CTX-M-15 among *Escherichia coli* and *Klebsiella pneumoniae*. Nevertheless, regional variations in the prevalence, circulating enzyme types, and sequence types (STs) among ESBL producers have been reported among different patient groups in different parts of the world (Bevan et al., 2017).

The Arabian Peninsula is a region with one of the highest prevalence rates of ESBL-producing enterobacterial species (Dandachi et al., 2019). In Qatar, the percentage of ESBL-producing *E. coli* and *K. pneumoniae* isolated from urine specimens in children in the outpatient setting increased from 18% in 2010 to 31.7% in 2018; the resistance was mainly due to CTX-M group 1 enzymes (89.2%; Eltai et al., 2018a). Recently, a study using a polymerase chain reaction fingerprinting approach revealed that 22.4% of diarrheagenic *E. coli* produced ESBLs, mostly CTX-M group 1 (*bla*_CTX__–__M__–__15_, *bla*_CTX__–__M__–__3_; Eltai et al., 2020). However, to the best of our knowledge, there are no epidemiological data regarding the STs of ESBL-producing Enterobacterales. As well, the molecular epidemiology of ESBL producers in children has never been studied using high-resolution genomics (Perez-Lopez et al., 2020). Whole-genome sequencing (WGS) has been demonstrated to be useful in antimicrobial resistance surveillance because of its sensitivity and accuracy to detect antimicrobial resistance genes or mutations (Hendriksen et al., 2019).

Our study aimed to use the WGS approach to comprehensively characterize ESBL-producing *E. coli* and *K. pneumoniae* recovered from screening and clinical specimens from pediatric patients at Sidra Medicine in Qatar. We also sought to determine to what extent the information generated by WGS analysis can help improve the clinical management of children with ESBL infections in our institution.

## Materials and Methods

### Study Design, Bacterial Isolates, and Screening Protocol

The study was conducted at Sidra Medicine, a new 400-bed tertiary care women’s and children’s hospital in Doha that has been functioning at full capacity since January 2018. The hospital serves as a referral center for the pediatric population of Qatar for all medical subspecialties, including oncology, neonatal intensive care and pediatric intensive care, and surgical specialties, including cardiac surgery and neurosurgery.

All non-duplicate ESBL-producing *E. coli* and *K. pneumoniae* isolates from clinical specimens as well as rectal swabs submitted for carbapenemase-producing organism (CPO) screening from children younger than 18 years with medical or surgical conditions that required an emergency or elective hospital admission were retrospectively studied for a 1-year period, between January and December 2018. When the same ST of an organism was recovered from both screening and clinical specimens, only the clinical strain was counted.

As a part of the infection control surveillance program in our institution, a CPO questionnaire-based risk assessment is performed on emergency and elective admissions. The questionnaire consists of three questions to identify previous colonization with multidrug-resistant organisms and admission and history of invasive procedures in the last 12 months in other health care facilities within or outside of Qatar. Rectal swabs are collected from patients who answered affirmatively to any of the questions or from those who are unsure about the answer to any of the questions. In addition, patients admitted to the neonatal intensive care unit (NICU) and pediatric intensive care unit (PICU) are routinely screened for CPO carriage on admission and then once a week.

### Bacterial Identification and Antimicrobial Susceptibility Testing

Screening rectal swabs were directly inoculated onto two selective chromogenic media (CHROMagar^TM^ ESBL and CHROMagar^TM^ mSuperCARBA, CHROMagar, France) to detect ESBL and carbapenemase producers, respectively. Colonies isolated from any of the screening media and Enterobacterales isolated from clinical specimens were identified using matrix-assisted laser-desorption ionization time-of-flight mass spectrometry (Bruker, Germany), Antimicrobial susceptibility testing for amoxicillin/clavulanate, piperacillin/tazobactam, ceftriaxone, ceftazidime, cefepime, aztreonam, ertapenem, meropenem, gentamicin, amikacin, ciprofloxacin, levofloxacin, cotrimoxazole, and nitrofurantoin was performed using the BD Phoenix^TM^ automated identification and susceptibility testing system (Becton Dickinson, United States). Minimum inhibitory concentrations (MICs) for colistin were determined by broth microdilution (ComASP^TM^ Colistin, Liofilchem, Italy). MIC and breakpoints were interpreted according to the Clinical and Laboratory Standards Institute (CLSI) guidelines 2018. Isolates with intermediate susceptibility and resistant to each antibiotic tested were grouped in a single non-susceptible category. The CLSI susceptible-dose-dependent category for cefepime was considered as intermediate for the purpose of this study. Non-susceptible isolates to any of the third-generation cephalosporins, cefepime, or aztreonam were considered as potential ESBL producers. Bacterial samples were stored at -80°C with 10% glycerol.

### Whole-Genome Sequencing and Bioinformatic Analysis

Genomic DNA was extracted using the automated platform NucliSENS easyMag (bioMérieux, Marcy-l’Etoile, France) and quantified by Qubit (Thermo Fisher Scientific, Waltham, MA). WGS was performed on all organisms with a susceptibility pattern consistent with ESBL production on the Illumina Miseq platform. Paired-end (PE) DNA libraries were constructed using Nextera XT (Illumina, San Diego, CA). The DNA was tagmented, amplified by index primers, and purified with AMPure XP beads (Beckman Coulter, Brea, CA) to remove the smaller-size fragments. DNA libraries were then normalized, pooled, and sequenced using the in-house MiSeq platform generation 300-bp PE reads (MiSeq Reagent Kit V3) at the Department of Pathology. Raw reads were assessed by Fastqc^1^ and quality trimmed by Trim Galore^2^ to eliminate adaptors and low-quality sequences. Trimmed reads were assembled *de novo* using SPAdes v.3.9.0 (Bankevich et al., 2012), and smaller contigs (<500 bp) were excluded. The assessment of all genome assemblies was performed using QUAST v5.0.2 (Gurevich et al., 2013), contaminant reads were excluded after analysis by Kraken v2 (Wood and Salzberg, 2014). STs and the genes encoding antimicrobial resistance for β-lactams, fluoroquinolones, aminoglycosides, and trimethoprim-sulfamethoxazole (cotrimoxazole) were predicted based on the PubMLST typing schemes implemented in *mlst*^3^ and the ResFinder v3.2 database (Zankari et al., 2012) implemented in *abricate* v0.9.8.^4^ Mutations in the quinolone resistance–determining regions (QRDR) affecting chromosomal genes encoding DNA gyrase and topoisomerase IV enzymes were characterized using AMRFinderplus v3.8 (Feldgarden et al., 2019). The plasmids were determined and defined based on the PlasmidFinder v2.1 database (Carattoli et al., 2014). The phylogroup and serotypes of *E. coli* were predicted using ClermonTyping (Beghain et al., 2018) and ECTyper.^5^ FimH typing was performed using^6^ (Roer et al., 2017). The genetic relatedness of the major STs in *E. coli* and *K. pneumoniae* was inferred based on core genome SNPs using Parsnp v1 (Treangen et al., 2014).

### Statistical Analysis

Associations were assessed by the χ^2^ test with Yates correction for continuity or Fisher exact test when an expected value was < 5. In addition, the analysis of some variables was broken down into three different nursing units based on the CPO screening policy: PICU and NICU, where active surveillance of ESBL carriage was performed on a weekly basis. The remaining units in which ESBL colonization were studied, depending on the result of the risk assessment survey, were grouped into general pediatrics (GPED).

## Results

A total of 327 ESBL producers were sequenced, 254 *E. coli* and 73 *K. pneumoniae*. Of them, 202 *E. coli* (79.5%) and 56 *K. pneumoniae* (76.7%) were recovered from screening specimens. Sixty-nine strains were isolated from clinical specimens [*E. coli* 52 isolates (25.5%) and *K. pneumoniae* 17 isolates (31.5%)], including urine [36 isolates (52.2%)], peritoneal fluid [8 isolates (11.6%)], respiratory tract [7 isolates (10.1%)], bloodstream [6 isolates (8.7%)], pus and wound [5 isolates (7.2% each)], and cerebrospinal fluid [2 isolates (2.9%)]. The proportion of clinical isolates in GPED, PICU, and NICU were 27.6% (55 isolates), 8.9% (8 isolates), and 15.8% (6 isolates), respectively (Supplementary Materials).

Overall, non-susceptibility rates among ESBL-producing *E. coli* and *K. pneumoniae* isolates were 46.1 and 72.6% for amoxicillin/clavulanate, 12.6 and 30.1% for piperacillin/tazobactam, 99.2 and 98.6% for ceftriaxone, 59.8 and 87.7% for ceftazidime, 68.5 and 74% for cefepime, 84.2 and 93.5% for aztreonam, 5.1 and 9.6% for ertapenem, 3.1 and 8.2% for meropenem, 18.1 and 30.1% for gentamicin, 0.8 and 4.1% for amikacin, 41.3 and 41.1% for ciprofloxacin, 42.4 and 17.9% for levofloxacin, 65.8 and 76.1% for cotrimoxazole, and 7.9 and 58.9% for nitrofurantoin. In addition, two *E. coli* strains isolated from rectal screening swabs were resistant to colistin (MIC between 4 and 8 mg/L). Except for the higher non-susceptibility rates to cefepime among clinical isolates of both species and higher non-susceptibility to nitrofurantoin among screening *E. coli* isolates, there were no significant antimicrobial susceptibility differences between clinical and screening isolates (Table 1).

**TABLE 1:** Comparison of non-susceptibility rates between clinical and screening ESBL-producing *E. coli* and *K. pneumoniae*.

	***E. coli***			***K. pneumonia***		
	**% Non-susceptible (no. isolates tested)**			**% Non-susceptible (no. isolates tested)**		
	**Clinical**	**Screening**	***p***	**Clinical**	**Screening**	***p***
CRO	100 (52)	99 (202)	0.4	100 (17)	98.2 (56)	0.6
CAZ	63.5 (52)	58.9 (202)	0.5	100 (17)	83.9 (56)	0.07
FEP	92.3 (52)	62.4 (202)	< 0.001	100 (17)	66.1 (56)	< 0.01
AZT	83.7 (49)	84.4 (122)	0.9	100 (17)	89.7 (29)	0.2
AMC	36.5 (52)	48.5 (202)	0.1	58.8 (17)	76.8 (56)	0.1
TZP	15.4 (52)	11.9 (202)	0.4	35.3 (17)	28.6 (56)	0.6
ERT	1.9 (1/52)	5.9 (202)	0.2	5.9 (17)	10.7 (56)	0.6
MEM	0 (52)	3.9 (202)	0.1	5.9 (17)	8.9 (56)	0.7
GEN	25 (52)	16.3 (202)	0.1	35.3 (17)	28.6 (56)	0.6
AMK	1.9 (52)	0.5 (202)	0.3	0 (17)	5.4 (56)	0.3
CIP	48.1 (52)	39.6 (202)	0.3	47.1 (17)	39.3 (56)	0.6
LVX	44.2 (52)	41.8 (184)	0.8	11.8 (17)	20 (50)	0.4
SXT	61.5 (52)	66.8 (202)	0.4	88.2 (17)	73.2 (56)	0.2
NIT	0 (52)	9.9 (202)	0.02	41.2 (17)	64.3 (56)	0.09

*CRO, ceftriaxone; CAZ, ceftazidime; FEP, cefepime; AZT, aztreonam; AMC, amoxicillin-clavulanate; AMS, TZP, piperacillin-tazobactam; ERT, ertapenem; MEM, meropenem; GEN, gentamicin; AMK, amikacin; CIP, ciprofloxacin; LVX, levofloxacin; SXT, trimethoprim-sulfametoxazole; NIT, nitrofurantoin.*

Ninety-seven different STs were detected in *E. coli*, whereas 40 STs were found in *K. pneumoniae*. The five most prevalent STs in *E. coli* were ST131 [43 isolates (16.9%)], followed by ST38 and ST10 [21 isolates (8.2% each)], ST1193 [10 isolates (3.9%)], and ST73 [8 isolates (3.1%)]. All ST131 isolates belonged to phylogroup B2. O25:H4 accounted for 54.8% of the 131 isolates. Twenty-two (51.2%) of the *E. coli* ST131 belonged to the subclone H30, and 13 (30.2%) of them to the clade H30Rx. Among *E. coli* ST131, non-susceptibility to fluoroquinolones was significantly higher in H30 subclone (90.9%; 20/22) compared with other subclones (47.6%; 10/21; *P* < 0.01). Among the other most prevalent *E. coli* STs, non-susceptibility to fluoroquinolones was particularly remarkable in ST1193 isolates (100%; Supplementary Materials).

The three most common STs among *K. pneumoniae* isolates were ST307 [7 isolates (9.7%)] followed by ST45 and ST268 [5 isolates (6.9% each)]. Non-susceptibility to fluoroquinolones was significantly higher in ST307 [85.7% (6/7)] than in other STs [36.4% (24/66)] (*P* = 0.02; Supplementary Materials). Phylogenetic analysis presented in Supplementary Figures S1, S2 indicates genetic variations among and within major STs in both species. For instance, two genetic groups were recognized in *E. coli* ST131 (Supplementary Figure S1B).

Eighty percent of NICU isolates, including all *K. pneumoniae* isolates, were detected during hospital stay. Carriage acquisition rate and infection acquisition rate in NICU during hospitalization for the study period were 3.28/1,000 and 0.82/1,000 patient-days. Twenty-nine percent of PICU isolates were detected during hospitalization. Carriage acquisition rate and infection acquisition rate in PICU were 5.82/1,000 and 0.76/1,000 patient-days. Cross-transmission of bacteria with the same ST between patients was not observed in critical care units. Overall, only three patients (4.3%) initially detected by screening swabs developed subsequent clinical infections caused by the same ST during the study period.

WGS revealed that all 327 isolates except one carried CTX-M genes. The predominant gene detected was *bla*_CTX__–__M__–__15_ in 287 isolates (87.8%), followed by *bla*_CTX__–__M__–__14_ and *bla*_SHV_-_106_ in nine isolates each (2.8%). ESBL-producing *E. coli* and ESBL-producing *K. pneumoniae* were isolated simultaneously from 17 samples with *bla*_CTX__–__M__–__15_ detected in both species in 16 of those samples.

Sixty-seven percent of isolates coproduced one or more β-lactamases. The most common genes were *bla*_TEM__–1B_ and *bla*_OXA__–__1_ in 134 (41%) and 64 (19.6%) isolates, respectively. CTX-M-15 was present in all isolates carrying *bla*_OXA__–__1_. In addition, 12 *E. coli* isolates and 6 *K. pneumoniae* isolates coproduced carbapenemases, Only two types of carbapenemases were detected: OXA-48 type (6 isolates) and NDM type (12 isolates; Table 1 and Supplementary Materials). Supplementary Table 1 shows the association between co-carriage of TEM-1B and OXA-1 and non-susceptibility to amoxicillin/clavulanate and piperacillin/tazobactam.

At least one plasmid-mediated quinolone resistance (PMQR) gene was detected in 199 isolates (60.9%). PMQR genes found were *qnr A/B/E/S* in 148 isolates (45.3%), *aac(6′)-Ib-cr* in 57 isolates (17.4%), and *qepA* in 2 isolates (0.6%). *oqxAB* was detected in one *E. coli* isolate only. Fifteen different aminoglycoside-modifying enzyme genes were detected. Ninety percent of isolates non-susceptible to gentamicin carried genes encoding AAC(3) enzymes (61/68 isolates). Conversely, only 2 (3.5%) of the 57 isolates harboring *aac(6′)-Ib-cr* were non-susceptible to amikacin. All four isolates with high-level resistance to amikacin carried 16S rRNA methylase genes (two *rmtB* and two *armA*). Genes encoding fosfomycin-modifying enzymes (*fos* genes) were detected in 73 isolates (22%). These genes were carried by 89% of *K. pneumoniae* and 3% of *E. coli* strains. The simultaneous carriage of *bla*_OXA__–__1_, *aac(6′)-Ib-cr*, and *aac(3)-II* was strongly associated (*P* < 0.0001). Eighty-nine percent of the isolates harboring the *dfrA17* gene also carried the *aadA5* gene, whereas all isolates harboring *dfrA12* carried *aadA2* genes (Table 2 and Supplementary Materials). The plasmid-mediated colistin resistance gene *mcr-1* was detected in the two colistin-resistant *E. coli* isolates (Supplementary Materials).

**TABLE 2:** Distribution of β-lactamase, quinolone, aminoglycoside, and cotrimoxazole resistance genes among ESBL-producing *E. coli* and *K. pneumoniae*.

	**GPED (199)**		**NICU (38)**		**PICU (90)**	
	***E. coli* (168)**	***K. pneumoniae* (31)**	***E. coli* (19)**	***K. pneumoniae* (19)**	***E. coli* (67)**	***K. pneumoniae* (23)**
**No (%)**						
**ESBL genes**						
*bla*_CTX__–__M__–__15_	142 (84.5)	31 (100)	19 (100)	18 (94.7)	56 (83.5)	21 (91.3)
*bla*_CTX__–__M__–__14_	8 (4.7)	—	—	—	—	1 (4.3)
*bla*_SHV__–__106_	—	6 (19.4)	—	2 (10.5)	—	1 (4.3)
*bla*_TEM__–__104_	5 (3)	—	—	—	3 (4.5)	—
*bla*_CTX__–__M__–__102_	5 (3)	—	—	—	3 (4.5)	—
*bla*_CTX__–__M__–__159_	5 (3)	—	—	—	2 (3)	—
*bla*_CTX__–__M__–__3_	2 (1.2)	—	—	—	1 (1.5)	—
*bla*_CTX__–__M__–__27_	1 0.6)	—	—	—	2 (3)	—
*bla*_SHV__–__27_	—	4 (12.9)	—	2 (10.5)	—	2 (8.6)
*bla*_CTX__–__M__–__1_	1 (0.6)	—	—	—	2 (3)	1 (4.3)
**Other βL genes**						
*bla*_TEM__–1B_	60 (35.7)	17 (54.8)	14 (73.7)	12 (63.2)	24 (35.8)	7 (34.8)
*bla*_OXA__–__1_	23 (13.7)	17 (54.8)	5 (26.3)	8 (42.1)	7 (10.4)	4 (17.4)
*bla*_*NDM 1/5*_	5 (3)	4 (12.9)	—	—	2 (3)	1 (4.3)
*bla*_*DHA*_	6 (3.6)	2 (6.5)	—	—	—	1 (4)
*bla*_TEM__–__33/35_	6 (3.6)	—	—	—	—	1 (4.3)
*bla*_OXA__–__48_	4 (2.4)	1 (3.2)	—	—	1 (1.5)	—
*bla*_*CMY*_	2 (1.2)	—	—	—	3 (4.5)	—
**PMQR-G**						
*aac(6′)-Ib-cr*	21 (12.5)	14 (45.1)	3 (15.8)	7 (36.7)	8 (11.9)	4 (17.4)
*oqxAB*	—	—	—	—	1 (1.5)	—
*qepA1*	—	—	2 (10.5)	—	—	—
*qnr A/B/E/S*	64 (38.1)	21 (67.8)	7 (36.8)	11 (57.9)	28 (41.8)	17 (73.9)
**AME-G**						
aac(3)-II*	28 (16.7)	11 (35.5)	5 (26.3)	8 (42.1)	11 (16.4)	1 (4.3)
*aadA1*	18 (10.7)	4 (12.9)	—	2 (10.5)	10 (14.9)	1 (4.3)
*aadA2*	6 (3.6)	6 (19.4)	6 (31.6)	2 (10.5)	5 (7.7)	5 (21.7)
*aadA5*	51 (30.4)	2 (6.5)	1 (5.3)	—	22 (32.3)	1 (4.3)
*aph(3′)-Ia*	13 (7.7)	4 (12.9)	—	2 (10.5)	2 (3)	4 (17.4)
*aph(3″)-Ib*	72 (42.8)	21 (67.8)	6 (31.6)	14 (73.7)	32 (47.8)	15 (65.2)
*aph(6)-Id*	69 (41.1)	21 (67.8)	6 (31.6)	14 (73.7)	33 (49.3)	16 (65.2)
**SXT-RG**						
*dfrA/B*	114 (67.9)	25 (80.7)	13 (68.4)	12 (63.2)	46 (68.7)	14 (60.9)
*sul1-3*	112 (66.7)	24 (77.4)	14 (73.7)	15 (78.9)	46 (68.7)	14 (60.9)

PMQR-G, plasmid-mediated quinolone resistance genes; AME-G, aminoglycoside-modifying enzyme genes; SXT-RG, cotrimoxazole resistance genes.*Comprised aac(3)-IIa and aac(3)-IId, aac(3)-IVa, and aac(3)-Via.

QRDR mutations (gyrA, parC, and parE) were detected QRDR mutations (*gyrA*, *parC*, and *parE*) were detected in 96.2% (101/105) of fluoroquinolone-non-susceptible *E. coli* isolates and 26.7% (8/30) of fluoroquinolone-non-susceptible *K. pneumoniae* isolates. Conversely, QRDR mutations were present in 30.2% (45/149) of fluoroquinolone-susceptible *E. coli* isolates and 7% (3/43) of fluoroquinolone-susceptible *K. pneumoniae* isolates. Overall, 69.8% (30/43) *E. coli* ST131 isolates carried ≥ 4 QRDR mutations compared with 27.5% (58/211) non-ST131 isolates (*P* < 0.001). Five identical QRDR mutations in *gyrA* (S83L and D87N), *parC* (S80I and E84V), and *parE* (I529L) were detected in 90.9% (20/22) of ST131-H30 isolates. Consistently, all *E. coli* ST1193 isolates had the same mutations in *gyrA* (S83L and D87N) and *parC* (S80I). All of the seven *K. pneumoniae* ST307 isolates carried two mutations in *gyrA* (S83I) and *parC* (80I) genes. In contrast, only two isolates (3%) belonging to other STs (ST967, ST2096) had two QRDR mutations (*P* < 0.001) (Supplementary Materials).

Narrow host-range IncF plasmids were present in 278 isolates (85%). FIB was the most common F replicon found in 243 isolates (74.3%) followed by FII in 163 isolates (49.7%) and FIA 99 isolates (30.3%). In addition, 10 more Inc replicon types were detected, mostly as a part of multiple combinations with F replicons. The three most prevalent were as follows: IncI1 [64 isolates (19.6%)], IncX [27 isolates (8.3%)], and IncB/O/K/Z [25 isolates (7.6%)]. On the other hand, 189 isolates (57.8%) contained col-type plasmids. RNAI [119 isolates (36.4%)], col156 [88 isolates (26.9%)], and MG828 [46 isolates (14%)] were the most common representative plasmids within this group. Plasmid location of *bla*_CTX__–__M_ genes could be determined in 22 isolates (Table 3). Twenty-one isolates (6.4%) did not have any plasmid detected. Both *mcr-1* genes found in our collection were carried by IncI2 plasmids (Supplementary Materials).

**TABLE 3:** Plasmid location of *bla*_CTX__–__M_ genes in 22 ESBL-producing *E. coli* and *K. pneumoniae* isolates.

Sample ID*	**Species**	**ST**	**Unit**	**Specimen**	***bla*_CTX__–__M_**	**Plasmid**
E6	*E. coli*	421	GPED	Rectal swab	CTX-M-15	IncFIB
E19	*E. coli*	73	GPED	Rectal swab	CTX-M-15	IncFII
CP28	*K. pneumoniae*	337	GPED	Rectal swab	CTX-M-15	IncFII
E35	*E. coli*	1193	PICU	Urine	CTX-M-15	IncFIA
E55	*E. coli*	131	PICU	Rectal swab	CTX-M-15	IncFIB
E58	*K. pneumoniae*	101	PICU	Rectal swab	CTX-M-15	IncFII
E60	*E. coli*	28	NICU	Rectal swab	CTX-M-15	IncFII
E102	*E. coli*	10992	GPED	Wound	CTX-M-15	IncI1
E128	*E. coli*	131	GPED	Rectal swab	CTX-M-15	IncB/O/K/Z
E157	*K. pneumoniae*	37	PICU	Rectal swab	CTX-M-15	IncI1
E172	*K. pneumoniae*	998	GPED	Rectal Swab	CTX-M-15	IncX1
E183	*E. coli*	69	GPED	Urine	CTX-M-15	IncFIA
E206	*K. pneumoniae*	10996	GPED	Rectal swab	CTX-M-15	IncFII
E215	*E. coli*	1611	GPED	Rectal swab	CTX-M-15	IncB/O/K/Z
E320	*K. pneumoniae*	133	PICU	Rectal swab	CTX-M-1	Col156
E360	*E. coli*	73	GPED	Urine	CTX-M-15	IncI1
E379	*K. pneumoniae*	37	PICU	Rectal swab	CTX-M-15	IncI1
E403	*K. pneumoniae*	37	NICU	Rectal swab	CTX-M-15	IncI1
E443	*E. coli*	773	GPED	Rectal swab	CTX-M-15	IncFIB
E486	*K. pneumoniae*	133	PICU	Rectal swab	CTX-M-15	Col156
E496	*E. coli*	517	GPED	Rectal swab	CTX-M-15	IncFIB
E497	*E. coli*	131	GPED	Rectal swab	CTX-M-15	IncFIB

*Sample code.

## Discussion

Our data show that CTX-M-type ESBLs are largely disseminated in the pediatric population in Qatar with CTX-M-15, the most widespread ESBL type worldwide (Bevan et al., 2017; Peirano and Pitout, 2019), as the main driving force. Our study also points out that narrow host range plasmids, mainly those belonging to the IncF family, are likely the main vehicle for the dissemination of *bla*_CTX__–__M__–__15_ along with other resistance genes such as *bla*_OXA__–__1_, *aac(6′)-Ib-*cr, and *aac(3)-II* among a great diversity of genotypes within *E. coli* and *K. pneumoniae* (Carattoli, 2009). It is reasonable to hypothesize that our epidemiological landscape has been shaped by a high rate of community carriage among a fast-growing expatriate population, which currently comprises around 87% of Qatar’s population. It is also important to note that the largest segment of the expatriate population in Qatar is constituted by a young labor force from the Indian subcontinent (De Bel-Air, 2019), where rates of CTX-M-15 among fecal isolates from healthy individuals and clinical isolates have been reported to be greater than 90% (Ensor et al., 2006; Lina et al., 2014; Chakraborty et al., 2015; Sherchan et al., 2015; Islam et al., 2019). In contrast, our NICU had a variety of genotypes of both species without subsequent significant cross-transmission, suggesting a sporadic colonization/infection within a group that is at particular high risk of ESBL acquisition (Li et al., 2017).

Sequence analysis revealed that the management of ESBL infections in our pediatric population is challenging, particularly concerning carbapenem-sparing options. The effect of clavulanic acid and tazobactam in inhibiting CTX-M enzymes is often counteracted in our isolates by the coproduction of TEM-1B and OXA-1 (Livermore et al., 2019) and to a lesser extent by other enzymes such as plasmid-mediated AmpC β-lactamases and carbapenemases. For example, amoxicillin/clavulanate does not seem to be a reliable agent for the treatment of uncomplicated ESBL lower urinary tract infections (UTIs). Fortunately, nitrofurantoin remains a good option in this scenario, especially in cases caused by *E. coli*, as all urinary isolates of *E. coli* were susceptible to this agent. On the other hand, although TEM-1B seems to have a poor inhibitory effect against tazobactam, the common coproduction of OXA-1 alone or accompanied by TEM-1B makes piperacillin/tazobactam an unreliable agent for the empirical or definitive treatment of systemic ESBL infections, irrespective of the unresolved controversy concerning the usefulness of this agent in high-inoculum infections (Retamar et al., 2013; Harris et al., 2018). Although ertapenem seems to be an appealing option, resistance to carbapenems due to coproduction of carbapenemases was not negligible. It was not surprising that OXA-48 type and NDM type, mainly associated with the coproduction of CTX-M-15, were the only carbapenemases identified in our strains, given that the molecular epidemiology of carbapenemases in *E. coli* and *K. pneumoniae* across countries of the Gulf Cooperation Council is largely dominated by these enzymes (Zowawi et al., 2014; Sonnevend et al., 2015; Dandachi et al., 2019; Touati and Mairi, 2020). Interestingly, the international high-risk clone of *K. pneumoniae* ST258 responsible for the dissemination of genes encoding KPC-type carbapenemases elsewhere was not detected in our collection concurring with earlier studies in this part of the Arabian Peninsula in which it was seldom found (Dandachi et al., 2019; Touati and Mairi, 2020). It should be also noted that 3 of 20 non-susceptible isolates to ertapenem lacked genes encoding carbapenemases or AmpC β-lactamases, suggesting that resistance to ertapenem might have been caused by impaired penetration through the outer membrane due to porin loss (Nicolas-Chanoine et al., 2018). Particularly noteworthy was high proportion of isolates susceptible to amikacin despite the presence *aac(6′)-Ib-cr* genes in 17% of strains. Although monotherapy with amikacin has been proposed to treat febrile UTI in children in regions with low resistance rates among ESBL producers (Madhi et al., 2018), it should be kept in mind that it is not uncommon to find susceptible isolates according to the CLSI breakpoints (MIC ≤ 16 mg/L) harboring *aac(6′)-Ib-cr* with MICs between 4 and 8 mg/L (Fernández-Martínez et al., 2015; Livermore et al., 2019). The treatment with amikacin of ESBL infections caused by isolates with MIC > 4 mg/L may become ineffective by compromising the C_max_/MIC ratio, the main pharmacodynamic parameter that predicts bacterial killing in aminoglycosides (Alqahtani et al., 2018). Likewise, resistance to cotrimoxazole among our isolates was significantly high, rendering this combination ineffective at least as empirical therapy. Interestingly, the strong association between *dfrA17* and *aadA5* genes and between *dfrA12* and *aadA2* genes suggests that class 1 integrons were not uncommon in our isolates. Class 1 integrons containing these gene cassette combinations are often located in IncF plasmids along with resistance genes such as *bla*_CTX__–__M__–__15_, *bla*_OXA__–__1_, and *aac(6′)-Ib-cr* (Oliveira-Pinto et al., 2017). Finally, the detection of *mcr-1* genes in two *E. coli* isolates resistant to colistin, the last resort for the treatment of Enterobacterales resistant to broad-spectrum cephalosporins and carbapenems, was worrying but not surprising as this gene has been sporadically reported in clinical specimens in the Arabian Peninsula carried by IncI2 and IncHI2 plasmids (Dandachi et al., 2019). It is worth noting that these isolates belonged to the STs ST115 and ST540, which have mainly been detected from the poultry and environment (Tsui et al., 2020). As fecal carriage of ESBL-producing *E. coli* has also been described from food handlers in Qatar (Eltai et al., 2018b), our findings suggest that a silent transmission from animals to humans via the food chain may be also playing a role in the spread of ESBL and other resistance determinants among our population.

Because fluoroquinolones are rarely prescribed in our institution, the high rate of non-susceptibility to these agents among a quinolone*-*naive population deserves a special mention. On the one hand, the high proportion of PMQR determinants in our isolates suggests that transmission of these genes among commensal intestinal flora through the epidemic plasmids reported here is facilitating the selection of isolates with multiple mutations in the QRDR resulting in high-level resistance to fluoroquinolones among community isolates (Yuan et al., 2012; Yassine et al., 2019). On the other hand, fluoroquinolone resistance has been a major driver for the selection and spread of successful clones that frequently harbor *bla*_CTX__–__M__–__15_ such as *E. coli* ST131-H30Rx, *E. coli* ST1193, and *K. pneumoniae* ST307 (Tóth et al., 2014; Johnson et al., 2015; Wyres et al., 2019; Fuzi et al., 2020; Peirano et al., 2020).

Our study has several limitations. Because MICs were determined by the Phoenix automated system, which does not always provide an exact value within the susceptibility range, low-level resistance conferred by certain genes could not be assessed when MICs did not exceed susceptible breakpoints, for instance, low-level amikacin resistance in isolates carrying *aac(6′)-Ib-cr* or low-level piperacillin/tazobactam resistance in the presence of OXA-1 enzyme. Moreover, the activity of other potential useful agents available in Qatar, particularly ceftazidime–avibactam, ceftolozane–tazobactam, and fosfomycin, could not be assessed because susceptibility cards used in this study did not contain any of these antibiotics. Despite this limitation, it could be speculated that ceftazidime–avibactam would have been effective against our isolates in the absence of coproduction of NDM-type carbapenemases, and fosfomycin would have been a suitable option for uncomplicated ESBL lower UTI caused by *E. coli* in the absence of other resistance mechanisms, as all isolates recovered from clinical specimens lacked *fos* genes (Cattoir and Guérin, 2018; Peirano and Pitout, 2019). It should be also taken into account that the population studied mostly comprised children at risk of acquisition of multidrug-resistant bacteria, which may have biased the actual burden of some resistance determinants in our entire pediatric population. Finally, plasmid replicons were analyzed using genome assemblies derived from short-read sequence data, which hindered reliable plasmid sequence reconstruction and elucidation of plasmid location of CTX-M genes in the majority of isolates. In fact, plasmid sequences were not detected by PlasmidFinder database in 22 isolates. Although this could indicate the challenge on the inference of plasmids from fragmented, small-sized contigs or the presence of non-typable plasmids, it could also suggest a potential chromosomal integration of *bla*_CTX__–__M_-type genes, a phenomenon increasingly reported in clinical isolates (Rodríguez et al., 2014; van Aartsen et al., 2019).

In summary, our study showed that CTX-M enzymes are overwhelmingly prevalent in the pediatric population of Qatar with CTX-M-15 as the major driving force. High community carriage rates among a multinational population appear to be the main reservoir for *bla*_CTX__–__M__–__15_ and other resistance genes, which are likely spread by epidemic plasmids. Our study also highlighted the promising value of NGS-based technologies to improve antibiotic prescribing practices by uncovering resistance mechanisms not detected by conventional phenotypic antimicrobial susceptibility testing. To the best of our knowledge, this is the first study characterizing ESBL in children using WGS in the Arabian Peninsula. Because demographic profiles in other countries of the Gulf Cooperation Council are similar to Qatar, we believe that our results could be extrapolated at least to the middle and eastern part of the region.

## Data Availability Statement

The original contributions presented in the study are publicly available. This data can be found in NCBI under accession numbers PRJNA599369 and PRJNA599387.

## Ethics Statement

This study was granted exempt status by the Institutional Review Board of Sidra Medicine (Protocol Number: 1804022140) as it only involved pathology specimens. Written informed consent for participation was not required for this study in accordance with the national legislation and the institutional requirements.

## Author Contributions

AP-L designed the study, analyzed the data, and drafted the manuscript. SS performed WGS, supported HA-M and MS in collecting isolates and performing antimicrobial susceptibility testing and revised the manuscript. HA-M supported MS in collecting isolates and performing antimicrobial susceptibility testing on selected isolates, supported SS in performing WGS, carried out the statistical analysis, and revised the manuscript. KT performed the bioinformatic analysis and revised the manuscript. MH supervised WGS process and bioinformatic analysis and revised the manuscript. MS performed identification and antimicrobial susceptibility testing on all isolates. EA and MJ collaborated with AP-L in the clinical interpretation of the WGS analysis. PT coordinated and supervised the execution of the study and substantially revised the manuscript. All authors read and approved the final version of the manuscript.

## Conflict of Interest

The authors declare that the research was conducted in the absence of any commercial or financial relationships that could be construed as a potential conflict of interest.

## Footnotes

1 http://www.bioinformatics.babraham.ac.uk/projects/fastqc/

2 http://www.bioinformatics.babraham.ac.uk/projects/trim_galore/

3 https://github.com/tseemann/mlst

4 https://github.com/tseemann/abricate

5 https://github.com/phac-nml/ecoli_serotyping

6 https://cge.cbs.dtu.dk/services/FimTyper/
