# Supplementary material for: Molecular Characterization of Extended-Spectrum β-Lactamase–Producing Escherichia coli and Klebsiella pneumoniae Among the Pediatric Population in Qatar
Source: Front Microbiol. 2020 Nov 11;11:581711. doi: 10.3389/fmicb.2020.581711 (PMC7686840; doi:10.3389/fmicb.2020.581711)
Supplement: Supplementary file 1 [file Presentation_1.PPTX]

## Slide 1
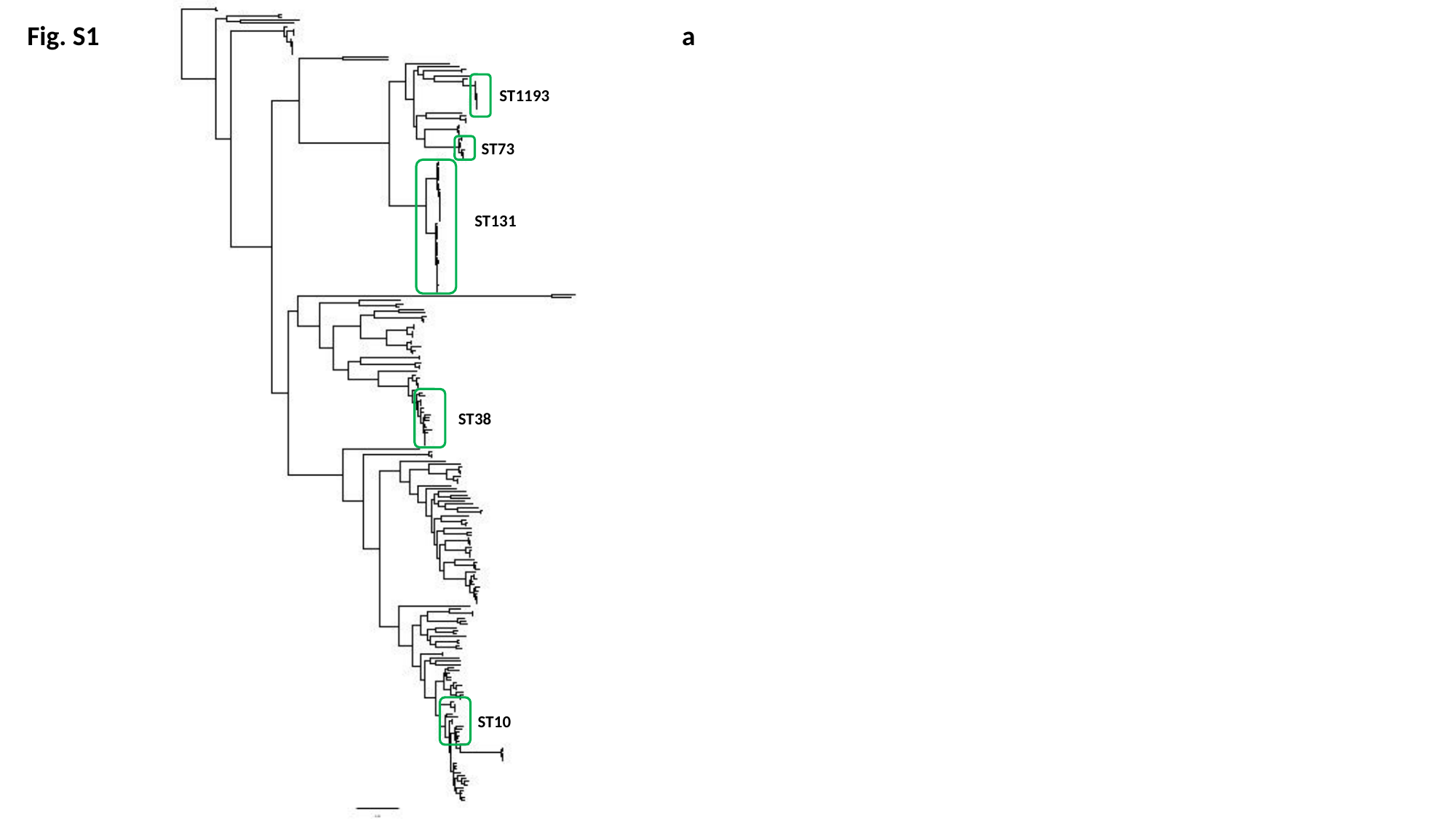

ST1193
ST73
ST131
ST38
ST10
Fig. S1
a

## Slide 2
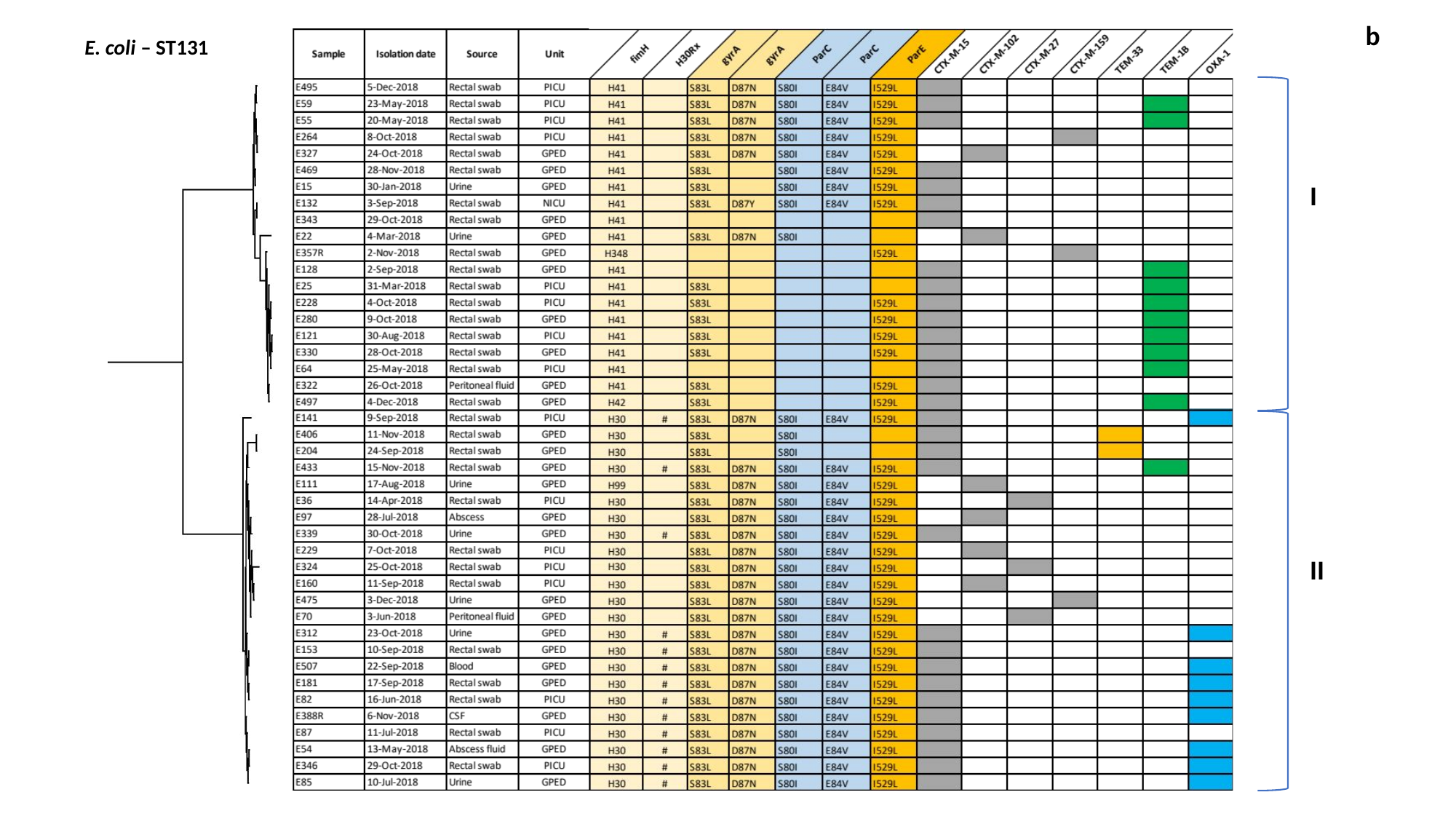

b
E. coli – ST131
I
II

## Slide 3
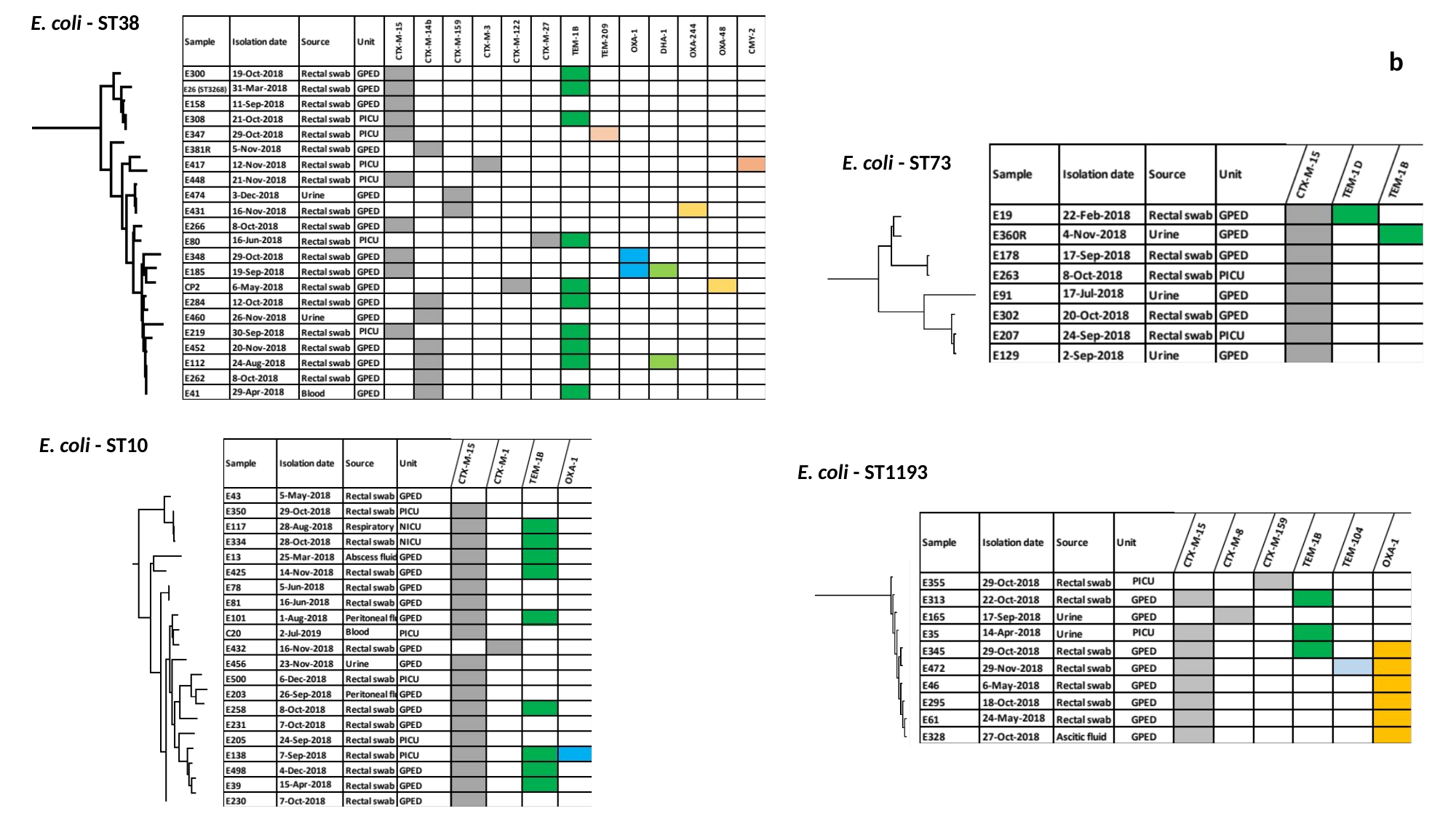

E. coli - ST38
b
E. coli - ST73
E. coli - ST10
E. coli - ST1193

## Slide 4
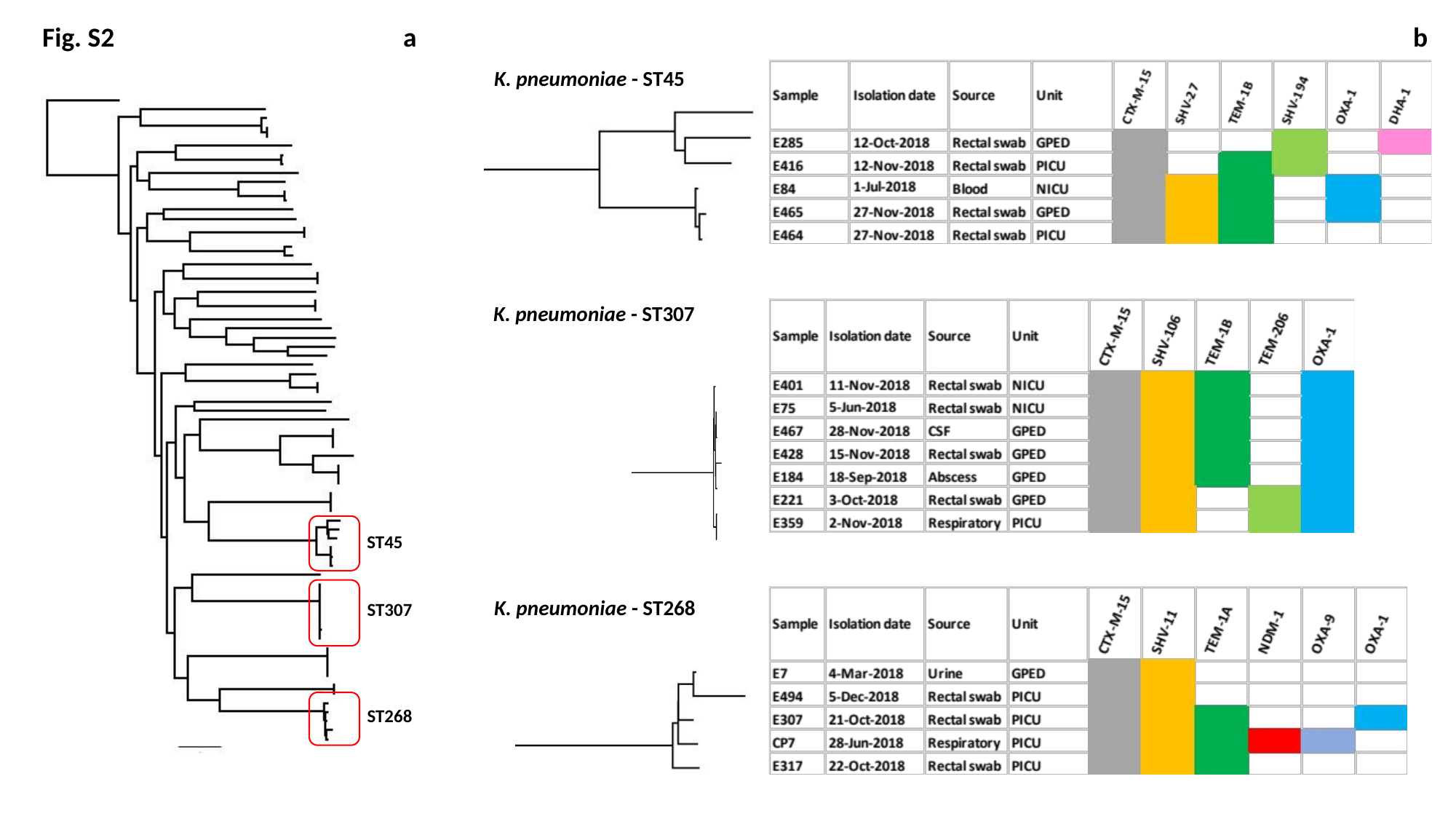

Fig. S2
a
b
K. pneumoniae - ST45
ST45
ST307
ST268
K. pneumoniae - ST307
K. pneumoniae - ST268
