# Supplementary material for: Molecular Characterization of Extended-Spectrum β-Lactamase–Producing Escherichia coli and Klebsiella pneumoniae Among the Pediatric Population in Qatar
Source: Front Microbiol. 2020 Nov 11;11:581711. doi: 10.3389/fmicb.2020.581711 (PMC7686840; doi:10.3389/fmicb.2020.581711)
Supplement: Supplementary file 3 [file Table_2.DOCX]

**Supplementary Table. Association between co-production of TEM-1B and OXA-1 and non-susceptibility to amoxicillin/clavulanate and piperacillin/tazobactam***

**Total** **AMC non-susceptible** **TZP non-susceptible**

**No. No. (%; *P* value) No. (%; *P* value)**

**TEM-1B only** 89 48 (53.9; <0.001) 5 (5.6; 0.5)

**OXA-1 only** 20 15 (75.0; 0.002) 5 (25.0; 0.02)

**Both together** 32 31 (96.9; <0.001) 15 (46.9; 0.004)

***Only isolates without co-production of other β-lactamases active against β-lactamase inhibitors, namely plasmid-mediated AmpC β-lactamases, (DHA-1, CMY 1/2/135), inhibitor-resistant TEM mutants (TEM 33/35) and carbapenemases (OXA-48-type and NDM-type), were analysed.**

**AMC: amoxicillin/clavulanate; TZP: piperacillin/tazobactam.**
